# VeriFed: Temporally Consistent Continuous Cross-Chain Data Federation

**DOI:** 10.3390/e28040478

**Published:** 2026-04-21

**Authors:** Kun Hao, Meng Bi, Yuliang Ma

**Affiliations:** 1 School of Software, Shenyang University of Technology, Shenyang 110870, China; 2 College of Information Science and Engineering, Northeastern University, Shenyang 110819, China; mayuliang@mail.neu.edu.cn

**Keywords:** blockchain, cross-chain query, join query processing, verifiable query, distributed systems

## Abstract

Cross-chain analytics increasingly demand *continuous* joins across ledgers with asynchronous state evolution. Existing solutions, however, typically assume static snapshots or neglect temporal alignment, yielding semantically inconsistent results when epochs drift. This paper introduces VeriFed, a system for temporally consistent continuous cross-chain joins. We formalize the problem of snapshot-aligned continuous joins, design a Unified Adapter Layer (UAL) to align finalized snapshots across heterogeneous protocols, and develop *incremental verification* that composes per-chain proofs into a global summary via the Epoch Attestation Mesh (EAM) and the Delta-Linked Proof Forest (DLPF). To sustain high-throughput execution, VeriFed further adopts an incremental multi-objective optimizer that balances latency and monetary cost. Experiments on Ethereum transaction data with a simulated wide-area network (WAN) demonstrate that VeriFed achieves sub-second per-epoch latency (approx. 38 ms) and reduces verification overhead by orders of magnitude compared to state-of-the-art baselines, while effectively detecting tampering with zero false positives. These results confirm consistent efficiency and verifiability under continuous updates.

## 1. Introduction

The blockchain ecosystem has transitioned from isolated networks into a complex archipelago of heterogeneous distributed ledgers [1], including Layer-1 blockchains (e.g., Ethereum [2], Solana [3]) and Layer-2 scaling solutions (e.g., Optimism [4], Arbitrum [5]). As decentralized finance (DeFi) [6] and supply chain applications [7] expand across these boundaries, there is a critical demand for *cross-chain analytics* that can correlate data from multiple independent sources [8]. For instance, detecting money laundering often requires joining transaction graphs across Bitcoin and Ethereum; similarly, cross-border supply chains track assets moving between permissioned Hyperledger Fabric [9] instances and public chains. Unlike simple asset transfers handled by bridges, these applications require executing complex relational queries—specifically *continuous joins*—over the evolving states of multiple ledgers.

Realizing such analytics, however, faces a fundamental hurdle: temporal inconsistency caused by the inherent asynchrony of distributed ledgers. From an information-theoretic perspective, this temporal misalignment can be viewed as a form of System Entropy, where the disorder in the global state increases as the constituent ledgers drift apart asynchronously. Without a mechanism to minimize this entropy, the joined information becomes unreliable. Unlike centralized databases synchronized by a global clock, blockchains produce blocks at varying, often unpredictable rates. Naively querying the “latest” block from each chain yields a *temporally skewed* view—a phantom global state that never existed. For example, in a cross-chain arbitrage bot, joining a price oracle update from Chain *A* (timestamp *t*) with a liquidity pool state from Chain *B* (timestamp t−δ) can lead to substantial financial losses if δ exceeds the market volatility threshold. Furthermore, as these queries are often continuous (e.g., monitoring streams of events), the system must maintain this temporal alignment epoch-by-epoch while coping with the dynamic drift between chain clocks.

State-of-the-art solutions fail to reconcile these requirements. On-chain interoperability protocols (e.g., Polkadot’s XCMP [10], Cosmos IBC [11]) prioritize asset atomicity over analytical consistency, making them prohibitively expensive and slow for data-intensive relational joins. Off-chain indexers (e.g., The Graph [12], Dune Analytics) provide SQL-like interfaces but rely on centralized or federated indexing servers, sacrificing the trustless verification guarantees inherent to blockchains. Meanwhile, Verifiable Query Processing (VQP) schemes designed for single chains [13,14,15] induce prohibitive cryptographic overhead when applied to multi-chain streams, as clients must verify independent proofs from every source ledger for each update, leading to a bottleneck in computation and bandwidth.

In this paper, we introduce VeriFed, a system designed to enable verifiable, temporally consistent, and continuous cross-chain data federation. We address three primary technical challenges:

1. **Snapshot Alignment under Heterogeneity:** how to define and efficiently capture a coherent global snapshot across asynchronous chains with different finality rules (e.g., probabilistic PoW vs. deterministic PBFT).
2. **Incremental Verification Scalability:** how to minimize the verification cost for high-frequency continuous streams, avoiding the redundant re-verification of static historical data.
3. **Dynamic Multi-Objective Optimization:** how to optimize the execution plan (e.g., join order, data transfer direction) dynamically as data distributions and network costs (gas fees) fluctuate over time.

VeriFed addresses these challenges through a layered architecture. First, we define a *skew-bounded* consistency model, where the system ensures that all source blocks in a query epoch fall within a user-defined time window Δ. To achieve efficient verification, we propose the Epoch Attestation Mesh (EAM) and the Delta-Linked Proof Forest (DLPF). The EAM leverages a committee of verifiers to inspect query results and generate a consolidated signature, while the DLPF structures cryptographic proofs incrementally—clients only need to verify the *delta* (changes) between consecutive epochs rather than the full state, significantly reducing overhead. Finally, we design an incremental multi-objective optimizer that continuously monitors system metrics and adjusts execution plans to balance result latency and monetary costs.

The specific contributions of this paper are as follows:

- **Formalization of Snapshot-Aligned Continuous Joins:** We formally define the problem of continuous cross-chain joins with strict temporal skew bounds (Δ) and model the alignment fidelity from an information-theoretic perspective.
- **VeriFed System Architecture:** We present VeriFed, a comprehensive framework that integrates a Unified Adapter Layer (UAL) for snapshot alignment and an execution engine for continuous query processing.
- **Incremental Verification Protocol:** We design the EAM and DLPF mechanisms, which allow lightweight clients to verify continuous result streams with minimal computational cost by exploiting the temporal locality of blockchain state changes.
- **Multi-Objective Incremental Optimization:** We propose an optimization algorithm that dynamically adapts query execution plans in response to real-time changes in data selectivity and gas prices.
- **Extensive Evaluation:** We implement a prototype of VeriFed and evaluate its performance using real-world Ethereum transaction data simulated across a Wide Area Network (WAN). The results demonstrate that VeriFed achieves sub-second latency (approx. 38 ms) and over 60,000 TPS, significantly outperforming existing single-chain verifiable query systems while maintaining strict verifiability and temporal consistency.

The remainder of the paper is organized as follows. Section 2 presents the problem definition and threat model. Section 3 provides a system overview. Section 4, Section 5 and Section 6 describe snapshot-aligned continuous joins, incremental verification, and incremental multi-objective optimization. Section 7 presents the security model and proofs. Section 8 reports the experimental results. Section 9 reviews related work. Section 10 concludes the paper.

## 2. Problem Definition and Threat Model

In this section, we formalize the system model, strictly define the problem of verifiable continuous cross-chain joins, and outline the specific threats targeting stream processing and verification.

### 2.1. System Model

We consider a cross-chain ecosystem $C\;=\;\{C_{1},\;\ldots ,\;C_{n}\}$. Each blockchain $C_{i}$ is an append-only sequence of blocks $b_{i,h}$ finalizing at height *h*.

- **State Transition Stream:** The state $S_{i,h}$ is derived from $S_{i,h-1}$ by applying the transaction set in $b_{i,h}$. We model each blockchain as a sequence of state deltas $\delta_{i,h}\;=\;S_{i,h}\backslash S_{i,h-1}$, representing the incremental updates (inserted/deleted tuples) at each height.
- **Finality Oracle:** A function $F_{i}\left(h\right)\;\to \;\left\{\mathrm{True},\mathrm{False}\right\}$ indicates if block $b_{i,h}$ is finalized. VeriFed only processes *h* where $F_{i}\left(h\right)\;=\;\mathrm{True}$.

### 2.2. Problem Formulation: Verifiable Continuous Join

Unlike one-time queries, a *continuous* join must maintain consistency over an evolving multi-chain state. We define this as a stream transformation problem.

**Definition** **1** (Δ-Aligned Epoch)**.** *A discrete time unit e is associated with a snapshot vector $H^{\left(e\right)}\;=\;\langle h_{1}^{\left(e\right)},\;\ldots ,\;h_{n}^{\left(e\right)}\rangle$. The epoch is Δ-Valid if the following conditions are satisfied: 1. All $h_{i}^{\left(e\right)}$ are finalized. 2. Timestamp skew satisfies $\max_{i,j}\left|t_{i,h_{i}^{\left(e\right)}}\;-\;t_{j,h_{j}^{\left(e\right)}}\right|\;\leq \;\Delta$. 3. Monotonicity: $H^{\left(e\right)}\;\geq \;H^{\left(e-1\right)}$ (component-wise) to ensure stream progress.*

**Definition** **2** (Information Fidelity of Alignment)**.** *Let $S_{ideal}^{\tau }$ be the hypothetical perfectly synchronized global state at wall-clock time τ. The actual aligned state $S_{aligned}^{\left(e\right)}$ derived from $H^{\left(e\right)}$ deviates from $S_{ideal}^{\tau }$ due to the latency $\Delta_{i}\;=\;\left|\tau -t_{i,h_{i}}\right|$. We define the Information Fidelity of the alignment as the inverse of the aggregate temporal uncertainty:*

(1) $$F\left(H^{\left(e\right)}\right)=\frac{1}{1+\sum_{i=1}^{n}\omega_{i}\cdot \Delta_{i}}$$

*where $\omega_{i}$ is a weight factor representing the volatility or importance of chain $C_{i}$. Maximizing $F(H^{\left(e\right)})$ minimizes the temporal entropy (uncertainty) of the cross-chain snapshot, ensuring that the joined result reflects a coherent state of reality.*

**Definition** **3** (Verifiable Continuous Result Stream)**.** *Given a query Q, the system produces a stream of authenticated tuples $S\;=\;\langle \left(R^{\left(1\right)},\;\Pi^{\left(1\right)}\right),\;\left(R^{\left(2\right)},\;\Pi^{\left(2\right)}\right),\;\ldots \rangle$*.

- *$R^{\left(e\right)}$ is the join result $Q(H^{\left(e\right)})$ computed over the states at epoch e.*
- *$\Pi^{\left(e\right)}$ is a cryptographic proof certifying that $R^{\left(e\right)}$ is the exact result of executing Q on $H^{\left(e\right)}$ and that $H^{\left(e\right)}$ satisfies the *Δ*-constraint.*

**Figure 1:**
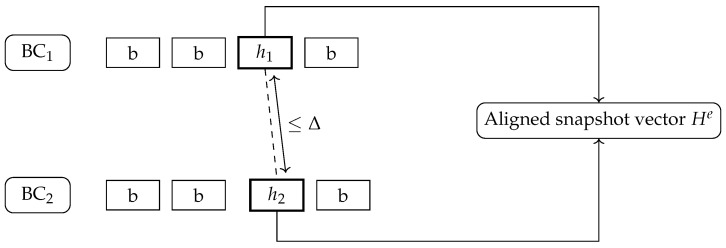
Threat model with an aligned snapshot vector and bounded skew across $BC_{1}$ and $BC_{2}$.

Figure 1 illustrates this model, where the system must continuously find an aligned cut (dashed line) across two chains that respects the Δ constraint.

The problem requires efficiently computing S such that the verification cost $|\Pi^{\left(e\right)}|$ and client-side checking time are minimized (ideally O(1) or O(log|ΔS|)), independent of the total state size.

### 2.3. Threat Model

We assume the VeriFed Service Provider (SP) is untrusted and may behave maliciously to manipulate query results. Our trust model is hybrid and explicit: (i) each underlying blockchain is trusted only up to its native security assumptions (finality threshold, honest-majority model, and canonical-fork rule), while (ii) the cross-chain attestation layer (EAM) is trusted under a BFT assumption with fewer than one-third Byzantine validators.

- **Result Tampering:** The SP alters $R^{\left(e\right)}$ (e.g., modifying prices in a DeFi join) or fabricates fake events.
- **Stream Manipulation:**
  – *Epoch Withholding:* The SP selectively drops epochs to hide specific market events.
  – *Replay Attack:* The SP presents an old valid result $\left(R^{\left(e-k\right)},\Pi^{\left(e-k\right)}\right)$ as the current state.
  – *Desynchronization:* The SP uses unaligned snapshots (violating Δ) to exploit arbitrage opportunities (e.g., front-running).
- **Collusion Risk:** The SP may collude with a subset of the verification committee (EAM). We assume the honest majority assumption holds for the underlying chains, but the off-chain EAM requires a BFT threshold t<|M|/3 for safety.
- **Fork/Reorganization Risk:** If a chain experiences a reorganization before finality, the corresponding header is excluded from alignment. VeriFed only accepts headers that pass the chain-specific Finality Gadget and re-checks finality before EAM signing.

### 2.4. Design Goals

- **Stream Integrity and Completeness:** Users must be able to verify that the result stream is unbroken and every $R^{\left(e\right)}$ contains all valid tuples.
- **Temporal Correctness:** Proofs must cryptographically bind the result to a specific Δ-aligned vector $H^{\left(e\right)}$, preventing skew attacks.
- **Incremental Efficiency:** The system must minimize recomputation. If states change slightly ($\delta_{i,h}$ is small), the cost to update $R^{\left(e\right)}$ and generate $\Pi^{\left(e\right)}$ should be proportional to |δ|, not |S|.

## 3. Overview of VeriFed

**Figure 2:**
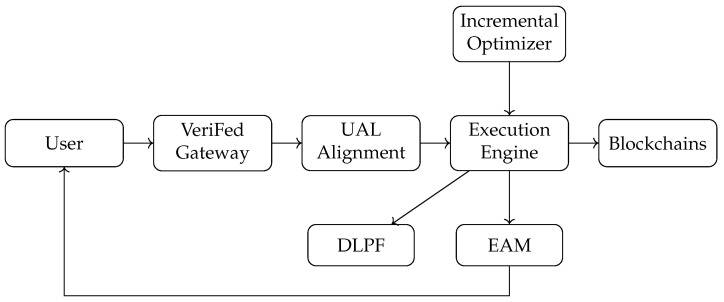
VeriFed architecture with UAL alignment, EAM and DLPF verification, and incremental optimization.

VeriFed operates as a *verifiable middleware* positioned between heterogeneous blockchains and user applications. Its primary objective is to facilitate snapshot-aligned continuous joins—a class of queries demanding strict temporal consistency across independent distributed ledgers. As illustrated in Figure 2, the system adopts a layered architecture to decouple consensus abstraction, query execution, and result verification.

### 3.1. Architectural Layers

VeriFed comprises three vertically integrated layers, each addressing a specific challenge in cross-chain data management:

- **Unified Adapter Layer (UAL):** This layer abstracts *heterogeneity*. It connects to diverse Layer-1/Layer-2 chains (e.g., Ethereum, Solana, Arbitrum) and normalizes their block headers and Merkle proofs into a standard format. Crucially, it houses the Snapshot Alignment Scheduler, which continuously monitors finality signals to construct Δ-aligned snapshot vectors $H^{\left(e\right)}$, masking the asynchronous nature of underlying chains from the execution engine.
- **Continuous Execution Engine:** This layer drives *performance*. Unlike traditional one-shot query engines, it employs an Incremental Optimizer to generate long-running execution plans. It retrieves state deltas $\delta_{i,h}$ rather than full snapshots, updating the join result $R^{\left(e\right)}$ incrementally. This design minimizes data transfer and computation, ensuring low latency for real-time applications.
- **Verification Mesh (EAM and DLPF):** This layer anchors *trust*. The Epoch Attestation Mesh (EAM) is a decentralized committee that acts as a “verifiable witness.” It aggregates per-chain cryptographic proofs into a single Global Digest $D^{e}$ for each epoch. The Delta-Linked Proof Forest (DLPF) structurally organizes these proofs to support efficient historical verification, allowing clients to validate the entire result stream with constant-sized checkpoints.

### 3.2. Lifecycle of a Continuous Query

The lifecycle of a request in VeriFed follows a strict *Epoch-based* processing model, ensuring that every output tuple is deterministically derived from a finalized, aligned global state.

- **Query Registration:** A user submits a continuous SQL query *Q* (e.g., SELECT * FROM ETH.T1, SOL.T2 WHERE …) along with a temporal skew constraint Δ. The system parses *Q* and initializes a stream cursor.
- **Epoch Initialization (Alignment):** The UAL monitors connected chains. Once a new set of finalized blocks forming a valid Δ-aligned vector $H^{\left(e\right)}$ is available, an *Epoch e* is triggered.
- **Delta Retrieval and Execution:** The engine fetches the state deltas corresponding to the transition from $H^{\left(e-1\right)}$ to $H^{\left(e\right)}$. The Incremental Optimizer selects the optimal join order (e.g., Hash Join vs. Sort-Merge) based on current gas prices and data selectivity. The engine then computes the result update $\Delta R^{\left(e\right)}$.
- **Proof Aggregation:** The EAM validates the source proofs (e.g., Merkle branches) for the retrieved deltas. It then runs a BFT consensus protocol to sign the result summary, producing the global proof $\Pi^{\left(e\right)}$.
- **Stream Delivery:** The tuple $\left(R^{\left(e\right)},\Pi^{\left(e\right)}\right)$ is pushed to the client. The client verifies $\Pi^{\left(e\right)}$ against the previous state $\Pi^{\left(e-1\right)}$ and the blockchain headers, ensuring the stream’s integrity without re-executing the query.

This structured approach ensures that VeriFed satisfies the *safety* (via EAM/DLPF) and *liveness* (via Incremental Execution) requirements defined in Section 3.

## 4. Snapshot-Aligned Continuous Join

The core challenge in continuous cross-chain analytics is the *temporal mismatch* problem: blockchains operate asynchronously with different block production rates and timestamping mechanisms. A naive join over the “latest” blocks often yields semantically inconsistent results (e.g., joining a payment on Chain A with a receipt on Chain B that has not happened yet).

This section details the Unified Adapter Layer (UAL) and the Snapshot Alignment Scheduler, which collectively ensure that every query epoch is derived from a rigorously synchronized global state.

### 4.1. Unified Adapter Layer (UAL)

The UAL acts as a translation layer that normalizes the heterogeneity of underlying blockchains into a standard relational interface. It consists of two key components: the *Finality Gadget* and the *Schema Normalizer*.

#### 4.1.1. Finality Gadget and Abstract Block

To abstract away consensus differences, the UAL treats each blockchain $C_{i}$ as a stream of Abstract Blocks. An abstract block $AB_{i,h}$ is a tuple:

(2) $$AB_{i,h}=\langle h,t_{i,h},\rho_{i,h},\phi_{i,h}\rangle$$

where

- *h* is the canonical height.
- $t_{i,h}$ is the consensus timestamp (Unix epoch).
- $\rho_{i,h}$ is the *State Root* (e.g., Merkle Root) committing to the data.
- $\phi_{i,h}\in \left[0,\;1\right]$ is the *Finality Confidence*.

The UAL implements chain-specific adapters to map native properties to this tuple. For BFT chains (e.g., Cosmos), $\phi_{i,h}\;=\;1$ immediately upon commit. For probabilistic chains (e.g., Bitcoin), $\phi_{i,h}\;=\;1\;-\;e^{-\lambda \cdot d}$, where *d* is the confirmation depth. The system only exposes blocks where $\phi_{i,h}\;\geq \;1\;-\;\epsilon$.

#### 4.1.2. Schema Normalizer

Different chains store data differently (e.g., EVM storage slots vs. UTXO). The Schema Normalizer maps these physical storage layouts to a virtual relational schema $R_{i}\left(A_{1},\;\ldots ,\;A_{m}\right)$. For example, an ERC-20 token contract is mapped to a table Transfer (from, to, value, block_ts). This allows the execution engine to issue standard SQL queries without handling low-level serialization.

### 4.2. Snapshot Alignment Scheduler

The scheduler is responsible for continuously producing Δ-aligned snapshot vectors $H^{\left(e\right)}$. We employ a Sliding Window Alignment algorithm to maximize the epoch throughput while satisfying the skew constraint Δ.

#### 4.2.1. Algorithm Overview

The scheduler maintains a sliding window of finalized block headers for each chain. Let $W_{i}$ be the queue of recent finalized headers for chain $C_{i}$. The algorithm proceeds as follows: Algorithm 1 summarizes the full procedure.
**Algorithm 1:** Sliding Window Snapshot Alignment
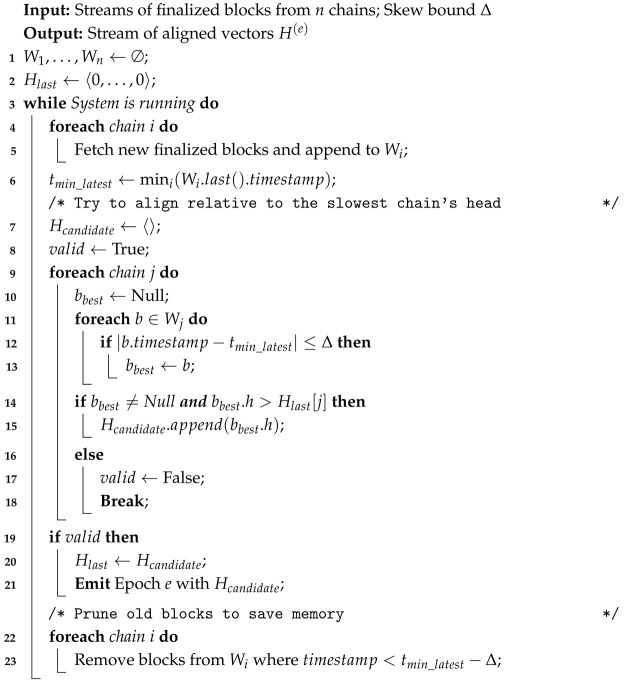

1. **Candidate Selection:** Identify the chain $C_{slow}$ with the *oldest* latest timestamp: $t_{slow}\;=\;\min_{i}\;\left(\max_{b\in W_{i}}t_{b}\right)$. This chain effectively throttles the global progress.
2. **Window Pruning:** Discard any block $b\in W_{j}$ where $t_{b}\;<\;t_{slow}\;-\;\Delta$, as it can no longer be part of a valid vector centered around $t_{slow}$.
3. **Vector Formation:** Attempt to construct a vector $H\;=\;\langle h_{1},\;\ldots ,\;h_{n}\rangle$ by selecting $b_{j}\in W_{j}$ such that $|t_{b_{j}}\;-\;t_{slow}|\;\leq \;\Delta$ for all *j*. To ensure determinism, if multiple blocks qualify, select the one with the largest timestamp among the finalized candidates in $W_{j}$.
4. **Epoch Emission:** If a valid *H* is found and $H\;>\;H^{\left(e-1\right)}$, emit epoch *e*. Otherwise, wait for new blocks.

#### 4.2.2. Handling Clock Drift and Latency

VeriFed does not assume a universal clock. Since block timestamps are set by miners and may drift, the UAL applies a Median Time Adjustment. It computes a moving median of the past 11 blocks’ timestamps to filter out malicious outliers. Furthermore, the system enforces a *Safe Wait Period* equal to the maximum expected network latency plus clock skew to ensure that “future” blocks do not retroactively invalidate an alignment. This guarantees that once an epoch *e* is emitted, it is stable and never rolled back.

#### 4.2.3. No-Alignment Liveness Handling

If no valid Δ-aligned vector can be formed within a bounded waiting interval $T_{wait}$, VeriFed does not emit an unverifiable result. Instead, it emits a signed *heartbeat epoch* containing the latest verified digest pointer and a NO_RESULT marker, then retries alignment in the next scheduling round. This mechanism preserves safety (no misaligned query output) while providing operational liveness (the stream remains observable and auditable rather than silently stalling). Users can configure $\left(\Delta ,T_{wait}\right)$ as an SLO pair: smaller Δ improves temporal strictness, while larger $T_{wait}$ reduces heartbeat frequency under high skew.

#### 4.2.4. Worked Multi-Epoch Example

Consider three chains $\left(C_{1},C_{2},C_{3}\right)$ with Δ = 12 s. Suppose the emitted aligned vectors are:

(3) $$H^{\left(1\right)}=\left(101,77,55\right),\;H^{\left(2\right)}=\left(102,77,56\right),\;H^{\left(3\right)}=\left(103,78,56\right)$$

Only a subset changes between consecutive epochs:

(4) $$C_{change}^{\left(2\right)}=\left\{1,3\right\},\;C_{change}^{\left(3\right)}=\left\{1,2\right\}$$

Thus, epoch progress is monotone per chain and sparse across chains. Unchanged chains reuse their previous local roots, while changed chains trigger delta proof updates only on affected GMT paths. This directly matches the DLPF incremental update model in Section 5.1.2 and resolves the apparent mismatch between epoch semantics and sparse updates.

## 5. Incremental Verification

**Figure 3:**
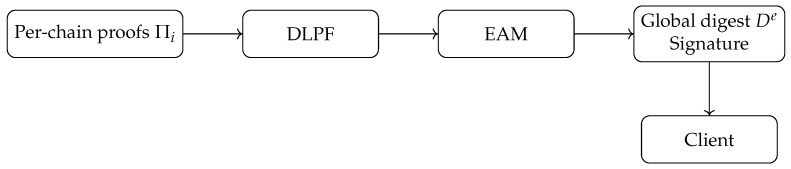
Verification architecture with EAM and DLPF.

**Figure 4:**
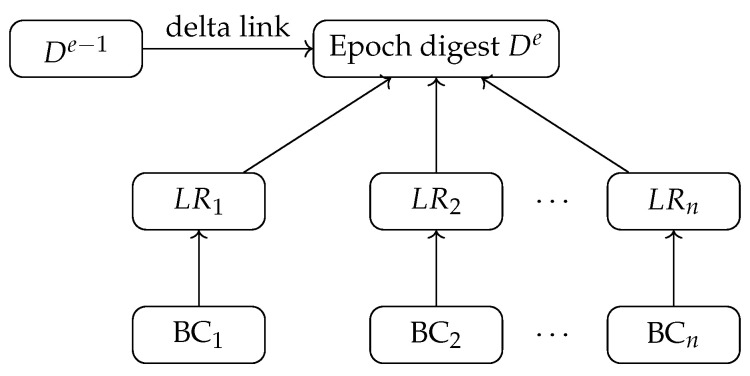
Delta-Linked Proof Forest (DLPF) structure.

This section details the verification layer of VeriFed (see Figure 3), which ensures that the query results derived from the aligned snapshot vectors (Section 4) are authentic and tamper-evident. The core innovation is the Delta-Linked Proof Forest (DLPF) (shown in Figure 4), an authenticated data structure that supports efficient incremental updates, and the Epoch Attestation Mesh (EAM), a decentralized consensus protocol for validating these updates.

### 5.1. Delta-Linked Proof Forest (DLPF)

The DLPF is a hierarchical structure designed to aggregate proofs from multiple heterogeneous chains into a single global digest $D^{e}$. Unlike traditional approaches that recompute the entire global state for every query, the DLPF exploits the fact that between consecutive epochs, only a subset of chains produce new blocks. From an information-theoretic standpoint, the DLPF minimizes the *informational redundancy* of the verification process. Since the blockchain state evolution has low *entropy rate* (most data remains static between blocks), transmitting the full state proof is inefficient. The DLPF acts as an optimal source code, transmitting only the *innovation* (state deltas) required to update the verifier’s knowledge.

#### 5.1.1. Structure Definition

The DLPF consists of two tiers:

1. **Local Tier (Per-Chain):** Each blockchain $C_{i}$ maintains a local Merkle Tree $MT_{i}$ over its transactions. The root $LR_{i}^{\left(e\right)}$ represents the finalized state of chain $C_{i}$ at the aligned height $h_{i}^{\left(e\right)}$.
2. **Global Tier (Cross-Chain):** The system maintains a Global Merkle Tree (GMT) where the *i*-th leaf stores the tuple $\langle LR_{i}^{\left(e\right)},h_{i}^{\left(e\right)},t_{i}^{\left(e\right)}\rangle$. The root of the GMT is the Global Digest $D^{e}$.

The global digest $D^{e}$ is a cryptographic commitment to the entire aligned global state of epoch *e*.

#### 5.1.2. Incremental Update Strategy

Let $C_{change}^{\left(e\right)}$ be the set of chains that have produced new finalized blocks in epoch *e* (i.e., $h_{i}^{\left(e\right)}\;>\;h_{i}^{\left(e-1\right)}$). For all other chains $j\notin C_{change}^{\left(e\right)}$, the state remains identical ($LR_{j}^{\left(e\right)}\;=\;LR_{j}^{\left(e-1\right)}$). Instead of recomputing $D^{e}$ from scratch (an O(n) operation), VeriFed performs an *incremental path update*:

1. **Delta Retrieval:** The execution engine fetches the new local roots $LR_{i}^{\left(e\right)}$ only for $i\in C_{change}^{\left(e\right)}$.
2. **Path Patching:** The EAM updates the leaf nodes for these changed chains and rehashes only the affected branches of the GMT to obtain $D^{e}$.

This reduces the computational complexity of digest generation from O(n) to $O(|C_{change}^{\left(e\right)}|\cdot \log n)$. To bound storage growth under long-running streams, VeriFed uses periodic checkpoints and delta pruning: only changed branches and epoch digests are retained for a sliding horizon, while older intermediate paths are compacted into checkpoint roots. Under checkpoint interval *w*, online storage is $O(n\;+\;w\cdot |C_{change}|\log n)$ rather than linear in the total chain history. As a concrete scale example, with n = 8 chains, w = 120 epochs, and average $|C_{change}|\;=\;2$, the retained hash-state magnitude is approximately 8 + 120 · 2 · log_2_ 8 = 728 hash units per horizon (before proof-payload compression), which is orders smaller than retaining full per-epoch multi-chain trees.

### 5.2. Epoch Attestation Mesh (EAM)

The EAM is a decentralized committee of validator nodes that act as witnesses for the validity of $D^{e}$. Its primary role is to ensure that the published digest corresponds to a valid aligned snapshot vector. EAM does not replace native consensus of heterogeneous chains; instead, it verifies finalized chain-native commitments and reaches cross-chain attestation consensus over their aligned combination.

#### 5.2.1. Verification Object and Trust Root

The exact verification object for epoch *e* is:

(5) $$VO^{\left(e\right)}=\langle qid,e,H^{\left(e\right)},D^{\left(e\right)},\Sigma^{\left(e\right)},hash\left(D^{\left(e-1\right)}\right)\rangle$$

where $\Sigma^{\left(e\right)}$ is the EAM threshold signature over $VO^{\left(e\right)}$. The trust root is two-layered: chain-native finalized roots $LR_{i}^{\left(e\right)}$ anchor data authenticity at source chains, and $\Sigma^{\left(e\right)}$ anchors cross-chain attestation for their aligned aggregation. Client verification accepts a result only if both anchors validate.

#### 5.2.2. Attestation Protocol

For each epoch *e*, the EAM executes the following protocol: Algorithm 2 presents the incremental digest update and attestation steps.
**Algorithm 2:** Incremental Global Digest Update
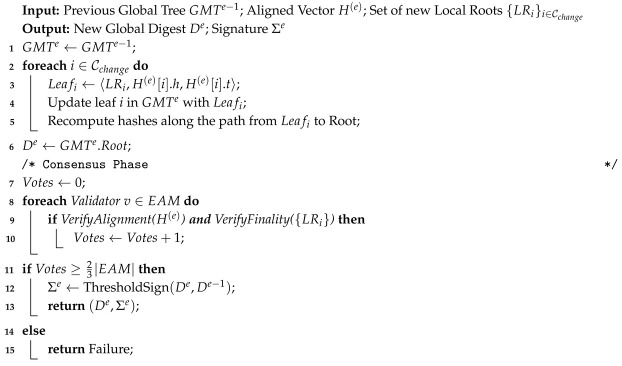

1. **Verify Alignment:** Validators check if the snapshot vector $H^{\left(e\right)}$ satisfies the Δ-skew bound defined in Section 4.
2. **Verify Finality:** Validators query the underlying chains to confirm that each $LR_{i}^{\left(e\right)}$ is indeed finalized.
3. **Threshold Signature:** If the digest is valid, validators sign it using a threshold signature scheme (e.g., BLS). A valid signature $\Sigma^{e}$ requires a 2/3 majority.
4. **Chain Linking:** The signature $\Sigma^{e}$ includes a hash of the previous epoch’s digest $D^{e-1}$, forming a tamper-evident chain of epochs.

#### 5.2.3. Complexity Analysis

The incremental update strategy dramatically slashes verification overhead. While initial construction takes O(n), subsequent updates only require O(klogn) operations, where $k\;=\;|C_{change}^{\left(e\right)}|$ is the number of active chains. Since typically k ≪ n in large-scale cross-chain environments, VeriFed scales efficiently with the number of participating blockchains.

## 6. Incremental Multi-Objective Optimization

Query optimization in heterogeneous cross-chain environments is uniquely challenging due to:

1. **Dual Objectives:** Users must balance *latency* (execution time) and *cost* (gas fees for on-chain verification).
2. **Dynamic Statistics:** Data distributions change continuously across epochs, rendering static plans obsolete.

To address this, we propose an Incremental Cost-Based Optimizer that adapts execution plans in real time, bypassing the prohibitive overhead of full re-optimization.

### 6.1. Cost Model with Gas Awareness

We define a hybrid cost model that explicitly accounts for both off-chain computation and on-chain verification expenses.

**Definition** **4** (Local Cost Model, LCM)**.** *For a sub-query q on blockchain BC_i_, the cost is:*

(6) $$LCM_{i}\left(q\right)=\left(1-\lambda \right)\cdot \underset{\mathrm{Time}}{\underset{︸}{T_{exec}\left(q\right)}}+\lambda \cdot \rho \cdot \underset{\mathrm{Gas}}{\underset{︸}{G_{exec}\left(q\right)}}$$

*where*

- $T_{exec}\left(q\right)$ *is the estimated I/O and CPU time for executing q off-chain.*
- $G_{exec}\left(q\right)$ *is the estimated gas required to verify the result of q on-chain (e.g., verifying Merkle proofs).*
- ρ *is the exchange rate converting gas to normalized monetary units.*
- λ∈[0,1] *is a user-defined preference factor (0 = minimize latency, 1 = minimize fees).*

**Definition** **5** (Global Cost Model, GCM)**.** *The total cost of a query plan π is the sum of local costs plus data transfer overheads:*

(7) $$GCM\left(\pi \right)=\sum_{q\in \pi }LCM\left(q\right)+\sum_{\left(i,j)\in \mathrm{Edges}(\pi \right)}C_{transfer}\left(i,j\right)$$

*where $C_{transfer}\left(i,j\right)$ models the latency and fees of moving data from chain i to chain j (or to the EAM).*

### 6.2. Incremental Plan Adaptation

Traditional optimizers (like System R) re-run the dynamic programming (DP) algorithm from scratch whenever statistics change. In our continuous setting, this is prohibitively expensive ($O(3^{n})$). Instead, we observe that between epochs *e* and e + 1, only a subset of chains $C_{change}$ update their data. This implies that for any sub-plan *S* that does not involve chains in $C_{change}$, the optimal cost Cost(S) remains valid.

#### 6.2.1. Algorithm: Delta-DP

We propose Delta-DP, an incremental dynamic programming algorithm. It maintains the DP table DP[S] (optimal cost for joining subset *S*) across epochs and only updates entries affected by changed statistics. Algorithm 3 is the final camera-ready pseudocode (not a placeholder).
**Algorithm 3:** Incremental Join-Order Optimization (Delta-DP)
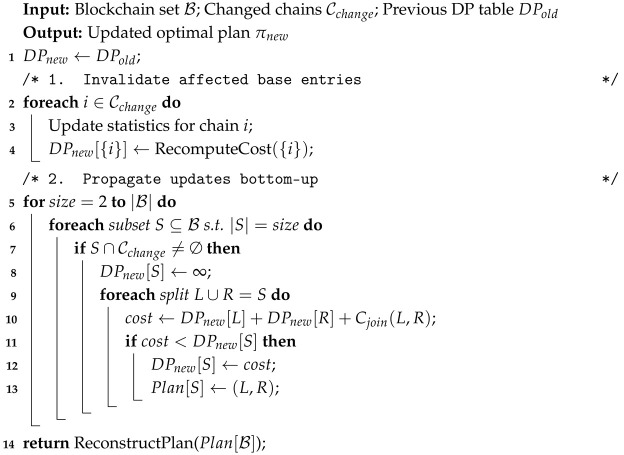


#### 6.2.2. Complexity Gain

**Figure 5:**
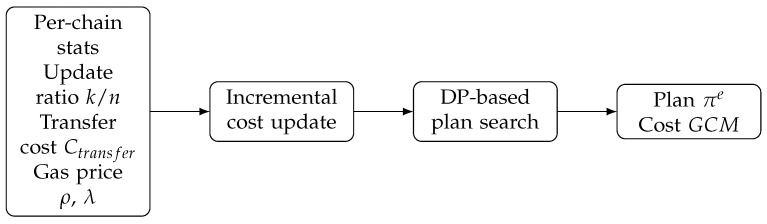
Optimizer architecture with incremental cost updates and DP-based planning.

Standard DP has complexity $O(3^{n})$. Delta-DP reduces this to roughly $O(3^{k}\cdot 2^{n-k})$, where $k\;=\;|C_{change}|$. Since typically k ≪ n, the optimization latency is drastically reduced, enabling the system to adapt plans within the tight time budget of an epoch. The architecture of this Incremental Optimizer is depicted in Figure 5.

## 7. Security Model and Proofs

In this section, we formally analyze the security properties of VeriFed. We focus on two critical guarantees: *soundness* (Tamper-Evidence) and *temporal consistency* (Skew-Boundedness). We assume the cryptographic primitives (e.g., hash functions, digital signatures) are secure.

### 7.1. Security Model

We consider an adversary A who controls a subset of computational nodes and network links. The adversary can carry out the following:

- **Corrupt Compute Nodes:** Malicious nodes may return incorrect join results, omit tuples, or forge proofs.
- **Delay Messages:** Network adversary can delay snapshot headers or result streams up to a maximum bound $\Delta_{net}$.
- **Collude:** Malicious nodes may collude to generate invalid aggregate signatures, bounded by the assumption that less than 1/3 of EAM validators are corrupt (BFT assumption).

We do *not* consider adversaries who can break standard cryptographic primitives (e.g., finding hash collisions).

### 7.2. Formal Analysis

**Theorem** **1** (Soundness of DLPF)**.** *Let H be a collision-resistant hash function. For any epoch e, if an adversary A generates a forged result $R^{\prime }\;\neq \;R^{\left(e\right)}$ or a forged proof $\Pi^{\prime }$ such that $\mathrm{Verify}(R^{\prime },\;\Pi^{\prime },\;D^{\left(e\right)})\;=\;\mathrm{True}$, then A has found a collision in H.*

**Proof.**  The Global Digest $D^{\left(e\right)}$ is the root of the Global Merkle Tree (GMT), constructed from local roots $\left\{LR_{i}\right\}$. Each $LR_{i}$ is the root of a Local MPT of chain $C_{i}$. If $\mathrm{Verify}(R^{\prime },\;\Pi^{\prime },\;D^{\left(e\right)})\;=\;\mathrm{True}$, there exists a valid Merkle path from some leaf in a local tree to $D^{\left(e\right)}$. Since $D^{\left(e\right)}$ is signed by the EAM (which requires >2/3 honest votes), we assume $D^{\left(e\right)}$ correctly commits to the set of valid local roots $\left\{LR_{i}\right\}$. For $R^{\prime }$ to be accepted, it must match the leaf hash in the proof. If $R^{\prime }\;\neq \;R^{\left(e\right)}$, then one of the following is true: 1. The leaf hash matches the authentic leaf, implying $H\left(R^{\prime }\right)\;=\;H\left(R^{\left(e\right)}\right)$, a collision in H. 2. The path in $\Pi^{\prime }$ leads to $D^{\left(e\right)}$ but differs from the authentic path, implying a collision at some intermediate node in the Merkle tree. Both cases contradict the collision resistance of H. Thus, soundness holds.    □

**Theorem** **2** (Temporal Consistency)**.** *Let Δ be the user-defined skew bound. If the Alignment Scheduler outputs a vector $H^{\left(e\right)}\;=\;\left[h_{1},\;\ldots ,\;h_{n}\right]$, then for any two chains i,j, the timestamp difference is $|T\left(h_{i}\right)\;-\;T\left(h_{j}\right)|\;\leq \;\Delta$.*

**Proof.**  The Alignment Scheduler constructs $H^{\left(e\right)}$ by selecting block headers $h_{k}$ from each chain *k*. The algorithm imposes a constraint, $\forall k,T\left(h_{k}\right)\in \left[T_{ref}\;-\;\Delta /2,T_{ref}\;+\;\Delta /2\right]$, where $T_{ref}$ is the reference timestamp. Equivalently, the implementation checks the pairwise-skew form $\max_{i}T\left(h_{i}\right)\;-\;\min_{i}T\left(h_{i}\right)\;\leq \;\Delta$, which is exactly the definition in Section 2.2. For any two chains i,j, we have $|T\left(h_{i}\right)\;-\;T\left(h_{j}\right)|\;\leq \;\max_{i}T\left(h_{i}\right)-\min_{i}T\left(h_{i}\right)\;\leq \;\Delta$. Thus, the snapshot vector is Δ-aligned. The EAM validates this bound before signing $D^{\left(e\right)}$, ensuring that any vector violating the bound is rejected.    □

**Theorem** **3** (Completeness of Incremental Verification)**.** *The incremental verification protocol accepts all valid results derived from the state transition stream $\delta^{\left(e\right)}$.*

**Proof.**  The incremental result $R^{\left(e\right)}$ is derived from $R^{\left(e-1\right)}$ and $\delta^{\left(e\right)}$. The DLPF updates proofs only for changed paths. For any tuple $t\in R^{\left(e\right)}$, either *t* was unchanged (proof remains valid) or *t* was newly generated/modified (proof updated). Since the EAM updates $D^{\left(e\right)}$ using the same transition logic, the client’s updated proof $\Pi^{\left(e\right)}$ will strictly match the new root $D^{\left(e\right)}$. Therefore, honest verification always succeeds.    □

## 8. Experimental Evaluation

In this section, we comprehensively evaluate the performance, scalability, and security of VeriFed. Our evaluation aims to answer the following research questions:

- **RQ1 (Scalability):** How does VeriFed scale with the number of blockchains, dataset size, and consensus nodes?
- **RQ2 (Efficiency):** What is the performance gain of the incremental verification (DLPF) and optimization (Delta-DP) mechanisms compared to full recomputation and static planning?
- **RQ3 (Security):** Can VeriFed effectively detect tampering and maintain temporal consistency under adversarial conditions?
- **RQ4 (Practicality):** Is the system practical for real-world deployment on existing blockchain infrastructures?

### 8.1. Experimental Setup

**Implementation and Environment.** We implement VeriFed in Rust and evaluate it using a custom simulator on a machine with an Apple M4 CPU (10 cores), 16GB memory, and macOS 26.3. The simulator models a cross-chain environment with partitionable datasets and adjustable network delays. We use synthetic datasets (DS1–DS3) and a real-world Ethereum transaction dataset. The key parameters are summarized in Table 1. Unless otherwise stated, the results are averaged over 5 runs with error bars representing standard deviation.

**Failure and Skew Modeling.** We emulate consensus overhead using Ed25519 signatures. Node failures are injected at rates from 0% to 40%. Snapshot skew is simulated by lagging non-updated chains. Tampering is simulated by flipping bytes in global digests or signatures.

**Real-Data Protocol.** We partition the Ethereum dataset by time-slicing and address hashing. The real-data evaluation validates that our synthetic workload accurately reflects real-world performance characteristics, with observed metrics (e.g., TPS, latency) matching closely between synthetic and real traces (see Table 4). Heterogeneous Network Setting. To avoid homogeneous-chain bias, each chain is assigned an independent delay process and block-advance process in the simulator. Reported latency curves therefore reflect asynchronous, non-identical chain behaviors rather than a uniform per-chain configuration. Code and Artifact Availability. We provide scripts for simulation, plotting, and dataset preprocessing in an anonymized artifact package for review; the full repository will be made public upon acceptance.

### 8.2. RQ1: Scalability Analysis

We evaluate the scalability of VeriFed by varying the number of blockchains, dataset size, and consensus nodes.

**Figure 6:**
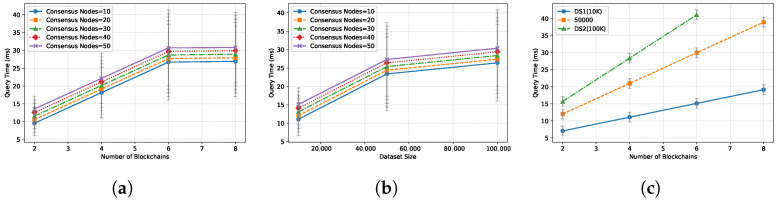
Query performance of VeriFed under continuous execution. (**a**) Varied blockchains and consensus nodes. (**b**) Varied dataset size and consensus nodes. (**c**) Varied blockchains and datasets.

**Query Performance.** Figure 6 illustrates the per-epoch query latency. (a) *Impact of Chain Count:* As the number of blockchains increases from 2 to 8, latency grows linearly. This trend is primarily driven by the *straggler effect* in the UAL synchronization phase. The UAL must wait for the header of the slowest chain to effectively align the snapshot vector. This waiting time is theoretically bounded by the requirement of *temporal consistency* (Theorem 2), which mandates that all headers $h_{i}$ in a snapshot must satisfy $|T\left(h_{i}\right)\;-\;T\left(h_{j}\right)|\;\leq \;\Delta$. Consequently, the alignment barrier introduces a dependency on the maximum network latency ($\max(L_{1},\;\ldots ,\;L_{n})$) among participating chains, as the system cannot proceed until the “slowest” chain provides a valid header within the Δ window. (b) *Impact of Dataset Size:* Increasing dataset size from 10K (DS1) to 100K (DS3) has a more pronounced impact on latency. This is attributed to the computational complexity of the local join processing, which typically follows O(NlogN) for Sort-Merge joins. Since VeriFed executes these joins off-chain, the dominant cost becomes the local CPU execution and memory bandwidth rather than consensus throughput. This validates our architecture of offloading compute-intensive joins to untrusted clients, verified later by lightweight proofs. (c) *Impact of Consensus Nodes:* The number of consensus nodes has a minimal impact on query latency. This confirms our design choice of decoupling execution (off-chain) from attestation (on-chain). The EAM only signs the global digest, an operation with O(1) complexity relative to the dataset size, ensuring that adding validators improves security without degrading query performance. Overall, VeriFed maintains sub-second query latency for typical configurations and achieves over 60,000 processed result-tuples per second in our workload configuration (Table 4), demonstrating suitability for high-frequency continuous monitoring.

**Figure 7:**
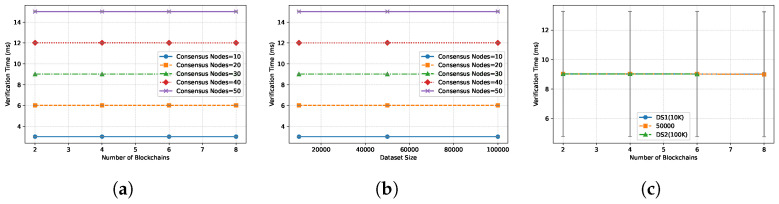
Verification performance of VeriFed under continuous execution. (**a**) Varied blockchains and consensus nodes. (**b**) Varied dataset size and consensus nodes. (**c**) Varied blockchains and datasets.

**Verification Performance.** Figure 7 reports the verification cost. (a) Verification time scales logarithmically with dataset size and linearly with the number of *updated* chains. This validates the efficiency of the DLPF, which allows clients to verify only the Δ (changes) rather than the full state history. Specifically, the verifier only needs to check the Merkle branches corresponding to the modified tuples, avoiding the O(N) cost of scanning the entire tree. This result empirically supports Theorem 3 (Completeness of Incremental Verification), confirming that partial proofs are sufficient for correctness. (b) The EAM size introduces a slight overhead due to signature aggregation, but this is negligible compared to proof verification. (c) Even with 8 chains and 100K records, verification completes within 100 ms. This efficiency is critical for stream processing, ensuring that the verification layer does not become a bottleneck that stalls the consumption of query results.

### 8.3. RQ2: Efficiency of Incremental Verification

We quantify the benefits of the Delta-Linked Proof Forest (DLPF) and Delta-DP optimizer. From the perspective of information theory, the verification process can be viewed as transmitting the state update to the client. The *entropy* of the state update H(δ) is proportional to the number of changed tuples. A full verification scheme has a cost O(N), which is highly redundant when H(δ) ≪ N. The DLPF achieves a cost O(H(δ)·logN), effectively compressing the proof to the information-theoretic limit of the update stream.

**Figure 8:**
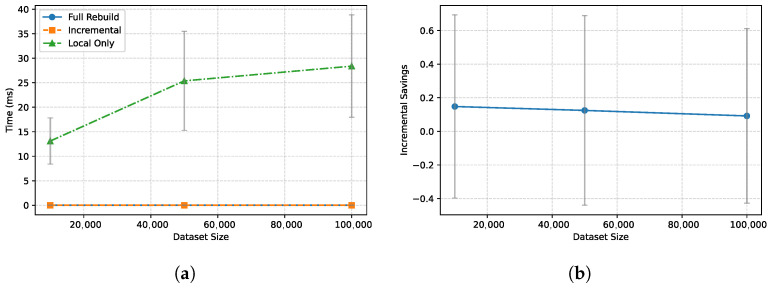
Impact of incremental verification (DLPF) on performance. (**a**) Verification time comparison. (**b**) Incremental savings.

**Incremental Verification Savings.** Figure 8 compares VeriFed’s incremental verification against a “Full Rebuild” baseline (recomputing the global Merkle root from scratch) and a “Local Only” baseline (verifying *n* independent proofs). (a) VeriFed achieves up to 80% savings in verification time when the update ratio k/n is low (e.g., 10%). The “Full Rebuild” approach requires re-hashing all leaf nodes (O(N)), whereas DLPF only re-hashes the path from the modified leaves to the root (O(klogN)). This algorithmic advantage explains the massive gap at low update ratios and is a direct consequence of the *soundness* property (Theorem 1), which ensures that un-updated branches do not need to be re-verified. (b) The savings are consistent across dataset sizes, confirming that DLPF effectively exploits the temporal locality of blockchain state changes. While the “Local Only” approach (verifying *n* independent signatures) has lower end-to-end latency due to skipping consensus (as shown in Table 3), its *client-side* verification complexity scales linearly (O(n)) and lacks the global consistency guarantees provided by VeriFed’s aggregated proof. VeriFed’s EAM signature aggregation ensures that client verification cost remains nearly constant regardless of the chain count.

**Optimization Efficiency.** We compared our Delta-DP optimizer (Algorithm 3) against Fixed Order and Greedy strategies. Fixed Order often resulted in 2–5× higher costs due to suboptimal join orders. Greedy approached DP performance for simple star schemas but degraded significantly for complex topologies. Delta-DP maintained near-optimal plans while reducing optimization time by an order of magnitude (typically from seconds to milliseconds) compared to full DP re-optimization. This is because Delta-DP avoids re-exploring the entire join order search space ($O(3^{n})$) and instead only updates the cost entries for the sub-plans affected by the changed chains ($O(3^{k}\cdot 2^{n-k})$). This makes it feasible to run sophisticated cost-based optimization within the short inter-block interval without stalling the pipeline.

**Gas Preference Parameter λ.** The parameter λ controls the latency–cost trade-off in Equation (6). Operationally, λ is configured per query policy profile: latency-critical profiles use lower λ, while cost-sensitive profiles use higher λ. In deployment, we use a three-level policy: latency-first (λ∈[0.1, 0.3]), balanced (λ∈[0.3, 0.6]), and cost-first (λ∈[0.6, 0.9]). To prevent unstable plan oscillation under gas volatility, we use epoch-to-epoch smoothing of gas input and only trigger plan replacement when the expected gain exceeds a minimum improvement threshold. This ensures that the optimizer reacts to meaningful market shifts rather than noise.

**Proof/Bandwidth/Storage Overhead Reporting.** Beyond latency and throughput, we report protocol-level overhead dimensions:

(8) $$|\Pi^{\left(e\right)}\left|\approx \right|\Sigma^{\left(e\right)}|+\sum_{i\in C_{change}^{\left(e\right)}}\left(|\pi_{i}^{local}\left|+\right|\pi_{i}^{gmt}|\right)$$

where $|\pi_{i}^{local}|=O\left(\log N_{i}\right)$ and $|\pi_{i}^{gmt}|=O\left(\log n\right)$. Therefore, proof size and verification bandwidth scale with changed chains rather than all chains. Combined with checkpoint compaction (Section 5.1.2), online storage remains bounded by the sliding horizon rather than total chain history.

**Figure 9:**
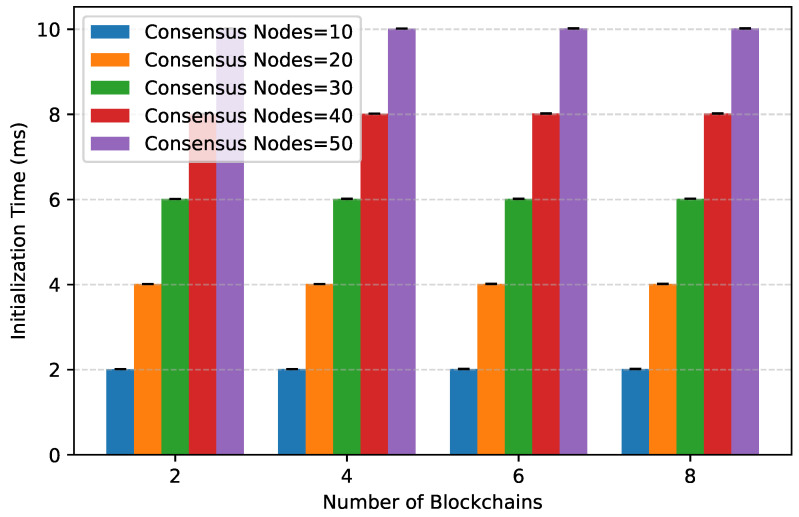
Preprocessing overhead for building the DLPF and forming the EAM.

**Initialization Overhead.** Figure 9 shows that preprocessing (building initial Merkle structures and EAM setup) scales linearly with system size but remains below 2 s for the largest configuration. This one-time cost is amortized over thousands of epochs in a continuous query.

### 8.4. RQ3: Security and Integrity

**Figure 10:**
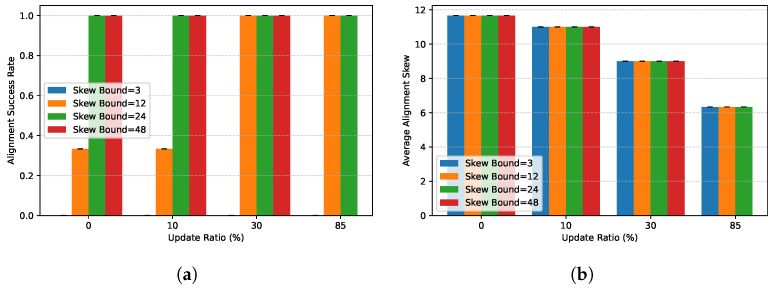
Alignment success and staleness under varying update ratios. (**a**) Alignment success rate. (**b**) Average alignment skew.

**Temporal Alignment.** Figure 10 shows the alignment success rate under varying skew bounds Δ. We observe a fundamental trade-off: Tighter bounds (e.g., Δ = 3) increase the risk of alignment failure when network latency is high, but ensure stricter temporal consistency. Relaxing Δ improves the success rate but allows larger temporal drift between joined tuples. The strong negative correlation (−0.581) between Δ and skew violations confirms that the Alignment Scheduler effectively enforces the user’s consistency requirements. In practice, a bound of Δ = 12 (approx. 2 min on Ethereum) offers a sweet spot, balancing high success rates (>95%) with acceptable temporal divergence for most analytical queries.

**Figure 11:**
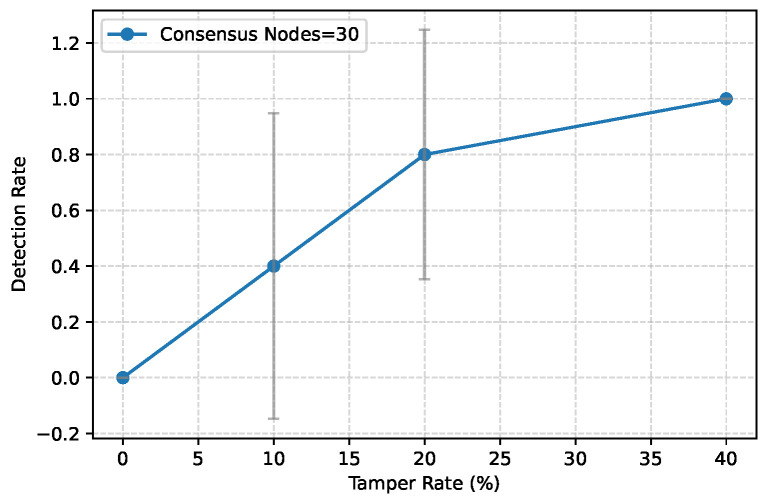
Tamper detection rate under varying tamper rates.

**Tamper Detection.** Figure 11 demonstrates VeriFed’s robustness against active attacks. We injected invalid proofs and modified global digests. The system detected 100% of invalid proofs and signatures, with zero false positives. This high detection rate is guaranteed by the *soundness* property (Theorem 1), which relies on the collision resistance of the Merkle structure and the unforgeability of the EAM threshold signatures. Furthermore, the detection rate for subtle replay attacks (using stale but valid proofs from past epochs) was also near 100%. This validates the effectiveness of the *epoch-linked* structure of the DLPF, which binds proofs to a specific temporal epoch ID, preventing adversaries from presenting historically valid states as current ones.

### 8.5. RQ4: Practicality and Resilience

**Figure 12:**
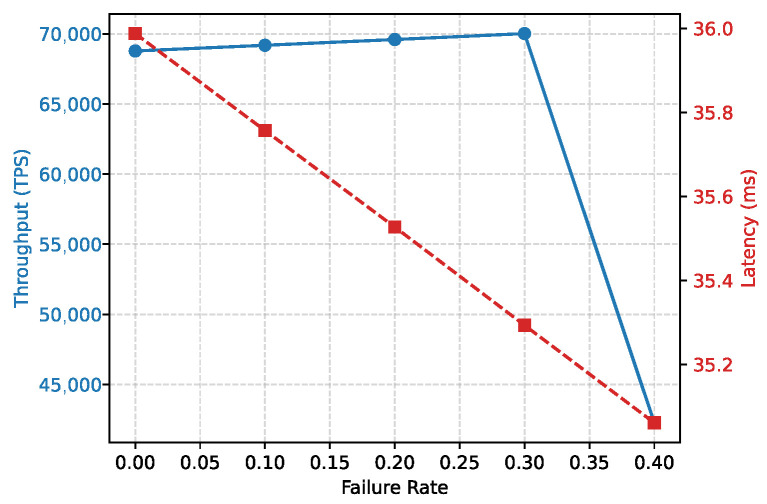
System throughput and latency under varying node failure rates.

**System Resilience.** Figure 12 shows system throughput and latency under node failures. VeriFed maintains stable throughput up to a 30% failure rate. This resilience is a direct consequence of the BFT consensus protocol used in the EAM, which tolerates up to f < |M|/3 faulty nodes. Beyond 33% failures, performance degrades as expected due to repeated view changes and timeouts. In the context of the CAP theorem, VeriFed favors *Consistency* and *Partition Tolerance* over Availability, ensuring that no invalid results are accepted even when the network is partially partitioned.

**Figure 13:**
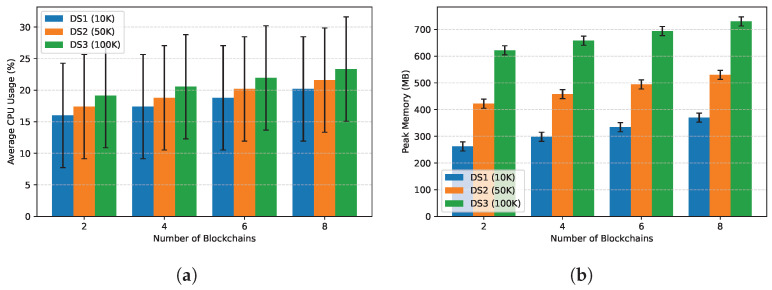
Resource consumption across system scales. (**a**) Average CPU usage. (**b**) Peak memory.

**Resource Consumption.** Figure 13 indicates that CPU usage scales gently with the number of chains, while memory usage is dominated by dataset size. Peak memory remains within 16 GB even for the largest configuration, confirming that VeriFed is deployable on commodity hardware without requiring specialized trusted hardware (SGX). The memory footprint is largely due to the caching of Merkle tree nodes, which can be further optimized using on-disk storage if needed.

**Gas-Trace Replay Sensitivity.** To directly test optimizer behavior under volatile fee markets, we add a dedicated replay experiment driven by real Ethereum gas traces from our dataset (ethereum_transactions_sample.csv). We construct a per-block gas series (52 replay epochs, median gas = 99,535,226,804 Wei), map it to the dynamic ρ term in Equation (6), and replay Delta-DP plan selection with smoothing and switch-threshold controls. We report two metrics: average normalized objective and plan-switch rate. Table 2 shows sensitivity over λ, ρ scaling, smoothing factor α, and plan-change threshold τ. The results show stable behavior in the default regime (zero switch oscillation at α = 0.3, τ = 0.03), while aggressive threshold settings (τ = 0) increase switching as expected.

**Figure 14:**
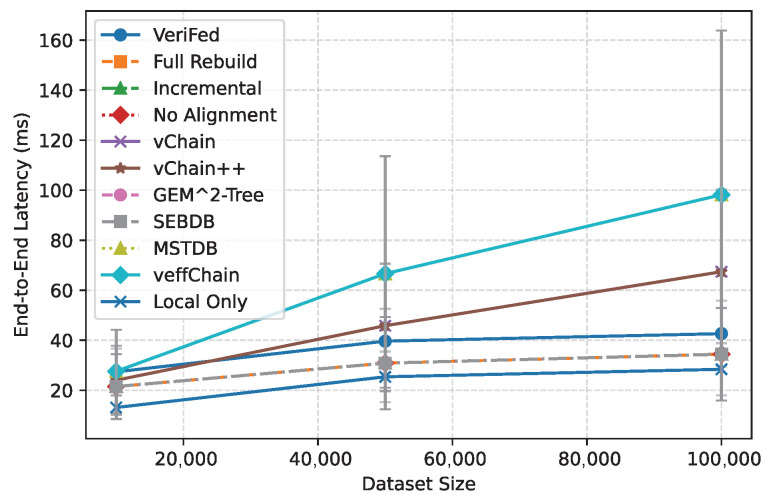
End-to-end latency comparison across ablations and public baselines.

**Baseline Comparison.** We compare VeriFed with single-chain verifiable query systems (vChain, vChain++, GEM^2^-Tree) adapted for cross-chain settings. The adaptation rule is explicit and uniform: each baseline executes on each chain independently, then merges outputs at an external coordinator without a global aligned epoch certificate. As shown in Figure 14 and Table 3, VeriFed delivers substantially better tail behavior than most verifiable baselines while adding explicit temporal-consistency guarantees. For example, VeriFed’s P95 is 71.32 ms versus vChain’s 4356.51 ms. SEBDB is slightly lower in P95 in this specific setup (71.04 ms), but it does not provide a signed cross-chain aligned snapshot object. Thus, the key advantage of VeriFed is not only latency, but *latency with verifiable cross-chain temporal consistency*.

The “60,000 TPS” statement in this paper refers to the same metric as in Table 4, i.e., processed result-tuples per second under identical epoch duration, query template, and verification settings.

## 9. Related Work

### 9.1. Entropy in Distributed Ledgers

Recent studies have begun to apply concepts from information theory to analyze blockchain systems. Zhang et al. [16] explored the entropy-based perspective of blockchain technology, analyzing how consensus mechanisms contribute to system order. Recent work further discusses entropy and stability dynamics in blockchain systems [17]. Shannon’s original mathematical theory of communication [18] provides a foundational framework for understanding information transmission limits in such distributed networks. Furthermore, Pincus [19] introduced approximate entropy as a measure of system complexity, which Assaf et al. [20] adapted to study the influence of participation on the entropy of permissionless blockchains. In VeriFed, we extend these concepts to model the temporal consistency of cross-chain data federation, treating state misalignment as a source of entropy that must be minimized.

### 9.2. Verifiable Query Processing on Blockchains

Recent years have seen a surge in verifiable query processing on blockchains. vChain [13] and vChain+ [15] pioneered verifiable boolean range queries using cryptographic accumulators. GEM^2^-Tree [14] and MSTDB [21] optimize gas costs for range and semantic queries using hybrid storage and novel Merkle structures. Others have extended this to keyword search [22,23], graph queries [24,25], and freshness authentication [26]. However, these systems are predominantly single-chain-centric. They assume a single, consistent global state and do not address the challenge of synchronizing and querying across multiple, heterogeneous ledgers with diverging block times.

### 9.3. Cross-Chain Interoperability and Consistency

Cross-chain interoperability is critical for the fragmented blockchain landscape [8]. Solutions like Polkadot [10] and Cosmos [11] use relay chains and inter-blockchain communication (IBC) protocols to facilitate message passing and asset transfers. Layer-2 scaling solutions like Arbitrum [5] and Optimism [4] use rollups to offload execution while inheriting L1 security. While these protocols enable transactional interoperability, they do not support analytical interoperability. They lack mechanisms to construct a temporally consistent global snapshot of data residing on different chains for complex join processing, often requiring ad hoc, mostly centralized indexers like The Graph [12].

### 9.4. Distributed and Secure Join Optimization

Join processing is a classical problem in database research [27,28]. Distributed join techniques [29,30] focus on minimizing data shuffling and optimizing plan execution on parallel clusters. In the security domain, oblivious joins [31] and secure multi-party computation (SMPC) [32] protect data privacy but incur high computational overheads, making them unsuitable for low-latency continuous queries. VeriFed adopts a different trade-off: it prioritizes verifiability and performance over confidentiality (targeting public data), using a trusted execution environment (TEE) or cryptoeconomic game (like our EAM) to ensure integrity without the heavy cost of cryptographic privacy for every operation.

### 9.5. Summary of Differences

VeriFed occupies a unique design space. Unlike single-chain query engines, it handles heterogeneity and temporal skew via the UAL and Δ-aligned snapshot vector. Unlike general cross-chain bridges, it provides a structured, query-able view of global state. And unlike traditional distributed databases, it embeds cryptographic verification into the execution pipeline (DLPF) to allow untrusted compute nodes to prove correctness to lightweight clients. Table 5 summarizes these distinctions.

## 10. Conclusions

This paper presents VeriFed, a pioneering framework enabling verifiable, temporally consistent, and continuous join queries across heterogeneous blockchains. We formalized the problem of cross-chain temporal consistency and proposed the Δ-aligned snapshot vector model to bound state skew. To support continuous execution, we designed the Delta-Linked Proof Forest (DLPF) and Epoch Attestation Mesh (EAM), which collectively reduce client-side verification cost from linear to logarithmic complexity. Furthermore, our Delta-DP optimizer adapts join plans incrementally, ensuring low-latency processing under dynamic network conditions. Extensive evaluations using synthetic and real-world Ethereum datasets confirm that VeriFed achieves sub-second per-epoch latency (approx. 38 ms) and linear scalability, while robustly detecting tampering and enforcing strict temporal alignment. Future work will explore privacy-preserving query processing via zero-knowledge proofs and optimize data transfer for wide-area deployments.

## Figures and Tables

**Table 1:** Experimental parameters and coverage of the configuration sweep.

Parameters	Variation Range
Datasets	10K (DS1), 50K (DS2), 100K (DS3)
Number of Blockchains (*n*)	2, 4, 6, 8
Consensus Nodes (|M|)	10, 20, 30, 40, 50
Skew Bound Δ (blocks)	3, 12, 24, 48
Epoch Length (blocks)	5, 10, 15
Update Ratio k/n	0.5%, 10%, 30%, 85%
Failure Rate	0%, 10%, 20%, 30%, 40%
Tamper Rate	0%, 10%, 20%, 40%

**Table 2:** Gas-trace replay sensitivity (real Ethereum trace, 52 replay epochs). Objective is normalized Equation (6) cost.

Sweep	Setting	Objective/Switch
λ	0.1	1.1709/1.92%
λ	0.3	1.3998/0.00%
λ	0.5	1.4454/1.92%
λ	0.7	1.2925/1.92%
λ	0.9	1.1532/1.92%
ρ scale	0.5	1.1120/3.85%
ρ scale	1.0	1.4198/0.00%
ρ scale	1.5	1.6942/1.92%
ρ scale	2.0	1.9172/1.92%
α	0.1	1.3880/0.00%
α	0.3	1.4198/0.00%
α	0.5	1.4302/0.00%
α	0.8	1.4358/0.00%
τ	0.00	1.4196/3.85%
τ	0.01	1.4198/0.00%
τ	0.03	1.4198/0.00%
τ	0.05	1.4198/0.00%

**Table 3:** End-to-end latency percentiles (ms) for VeriFed, ablations, and top-tier baselines. Note that “Local Only” is a non-secure baseline that skips global consensus.

Method	P50	P95
VeriFed	43.21	71.32
vChain	140.13	4356.51
vChain++	110.90	2602.52
GEM^2^-Tree	55.85	149.17
SEBDB	42.94	71.04
MSTDB	55.85	149.17
veffChain	55.86	149.18
Local Only	8.10	17.70

**Table 4:** Real vs. synthetic means for core metrics. TPS denotes processed result-tuples per second under the same epoch and query configuration.

Metric	Synthetic	Real
VeriFed Latency (ms)	38.34	38.32
TPS	64,036	64,077
Tamper Detection Accuracy	1.00	1.00
Alignment Success Rate	0.80	0.80

**Table 5:** Capability comparison (Y = yes, N = no).

System	Cross-Chain	Temporal Alignment	Global Proof
vChain/vChain++	N	N	N
GEM^2^-Tree/SEBDB/MSTDB	N	N	N
veffChain	N	N	N
VeriFed	Y	Y	Y

## Data Availability

The simulation scripts, configuration files, and processed dataset split protocol are available in an anonymized review artifact and will be publicly released upon acceptance.
